# News and Coming Events

**Published:** 1909-03-27

**Authors:** 


### News and Coming Events.

The new out-patient department of the Royal Boscombe and West Hants Hospital will be opened by H.R.H. the Duchess of Albany on Saturday, April 3.

With reference to the outbreak of smallpox in London, the Metropolitan Asylums Board announce that there have been four cases since February 4, one of which proved fatal. The last of these was received on March 17. The first of the four cases came from Camberwell, and the others occurred in St. Giles, Hackney, and Westminster. Until the first of these cases London had been free from any visitation of smallpox for fifteen months. The present cases are regarded by the medical officers to the Board as sporadic.

The Eighth International Congress of Hydrology and Climatology, including geology and physical therapeutics, will be held this year at Algiers from April 4 to 10, under the patronage of the Governor-General of Algeria. The last meeting of the Congress was at Venice four years ago. The local secretaries for Great Britain are Dr. G. Pernet and Dr. Purves Stewart, 94 Harley Street, London, W. An exhibition will be held in connection with the Congress.

The Duke of Wellington presided at the annual meeting of the Brompton Consumption Hospital on March 19. Lord Cheylesmore moved the adoption of the report for 1908, which shows an increase in the grants to the hospital from the King's Fund and the Hospital Sunday Fund of £3,500 and £3,420 respectively. The committee regard this as in a large measure due to the remarkable success which continues to result from the special form of treatment by graduated exercises carried out at the Frimley Sanatorium. The patients in the hospital on January 1, 1908, numbered 306. During the year 1,367 were admitted and 82 deaths occurred.

The annual council of governors of the Great Northern Central Hospital was held on March 18 under the presidency of Sir John Dickson-Poynder, M.P. (chairman of the committee of management). The chairman stated that there was a deficiency on the past year of £4,643, and at the present time an indebtedness to the bankers of £10,304. It would be a calamity to the poor of Islington if a ward should have to be closed, and, moreover, such a course, which will have to be adopted unless substantial help is soon forthcoming, would be a serious reflection upon the borough.

On March 18, in the House of Commons, Captain Craig asked the First Commissioner of Works whether he would consider the advisability in the public interest of altering the regulations regarding the Royal parks in order to permit members of the medical profession to pass through during the prohibited hours in motor-cars, whether petrol or electric driven; and whether special permits for this purpose could be issued. Mr. Harcourt replied that Hyde Park is the only Royal park closed to petrol-driven motors during three hours of the afternoon for the three months May to July. As the speed of motors in the parks is limited to ten miles, whilst the street limit is 20 miles, members of the medical profession in a hurry to reach their patients and to avoid penalties would be well advised during these and all other months to avoid the parks and stick to the streets.

H.M. the King of Roumania has given a further decoration to Mr. Edward D. Madge, M.R.C.S., by the bestowal of the Order of the Star of Roumania upon him.

H.M. the King of the Hellenes has decorated Mr. H. Stanley Turner, M.R.C.S., with the Knighthood of the Royal Order of the Saviour.

General Sir J. Ramsay Slade, R.A., has been elected Chairman of the Committee of Management of the Italian Hospital, Queen Square, W.C.

Surgeon-General George D. Bourke, C.B., has been appointed an honorary physician to the King, *vice* Surgeon-General T. Tarrant, C.B., deceased.

Dr. Bonnje, senior medical officer of the Russian Squadron which was entertained last week in London by the Admiralty, and seven other Russian medical officers lunched with Inspector-General T. D. Gimlette and the officers of Haslar Hospital on March 17. The Russian officers were afterwards shown over the hospital.

Dr. G. F. Buchan, M.B., D.P.H., deputy medical officer for the city of Birmingham, has been appointed medical officer of health for the district of Heston and Isleworth, in Middlesex, in succession to Dr. E. Steigman, who is secretary to the Commission on Tuberculosis.

The report of the Royal Dental Hospital of London, Leicester Square, presented on March 11 to the annual meeting, states that the number of patients last year was 20,405, the number of attendances 57,960. There was an increase of nearly £100 in the annual subscriptions. An X-ray department, installed at a cost of £200, was provided by the " A. J. Woodhouse " legacy. The year closed with a deficit of £905. The annual meeting was presided over by Mr. Morton Smale, a vice-president and consulting dental surgeon. He referred to the fact that 1908 was the jubilee year of the hospital, and a real effort had been made to pay off some of the debt. In response to an appeal issued by Sir Frederick Treves and in virtue of the £1,000 from the King's Hospital Fund, the hospital debt had been reduced to £43,000. In twenty-three years that debt must be paid.

The Prince and Princess of Wales paid a surprise visit of two hours' duration to the Middlesex Hospital on the afternoon of March 22; and, accompanied by Prince Francis of Teck, a Vice-President of the hospital, were conducted over the institution by the Secretary-Superintendent, the Lady Superintendent, and the Resident Medical Officer. The wards, chapel, nurses' refectory, and the kitchens were all inspected; and the Royal visitors displayed particular interest in the Princess May ward for children, and in the new maternity wards recently established at the hospital, with the arrangements of which they expressed great satisfaction. A visit was also paid to the medical school and the museum, and to the special wing for incurable cancer patients. Finally, the Cancer Research Laboratories were inspected, and the Prince and Princess showed the greatest interest in the investigations—especially those in connection with radio-activity in cancer—which were being carried out, the lines of which were explained to them by Dr. Lazarus-Barlow, the director.

The 136th Anniversary Dinner of the Medical Society of London, organised by the Hon. Secretaries, Mr. J. H. Kellock and Dr. Leonard Guthrie, was held on March 10 at the Hôtel Métropole under the chairmanship of Mr. C. B. Lockwood, F.R.C.S., the President of the Society for the current year. The financial position of the Medical Society was announced by the Chairman to be highly satisfactory. Among the other speakers were Sir Wm. Church, President of the Royal Society of Medicine; Sir Alfred Keogh, who paid a high compliment to the medical profession for its response to the needs of the Territorial Medical Service; and Dr. J. Kingston Fowler, last year's President of the Medical Society.

---

The sixty-ninth annual meeting of the Metropolitan Convalescent Institution was held last week, with Mr. M. O. Fitzgerald in the chair. Among those present were Viscount Clifden and Mr. D'Arcy Power, F.R.C.S. The Chairman stated that the laws relating to the property of the Institution had been revised in order to allow the invested funds and the real estate of the charity to be transferred to the "Official Trustees of Charitable Funds" and the "Official Trustee of Charity Lands" respectively, to be held by them in trust for the Institution. The number of patients admitted during 1908 was 7,526, and the average number of beds occupied daily throughout the year at the four homes was 401. The number of patients admitted to the wards set apart for surgical convalescents was 637. An urgent appeal is made for the £7,000 required for the completion of the seaside branch for men at Little Common, Bexhill-on-Sea.

---

Mr. Stephen Paget, F.R.C.S., on March 18 delivered a lecture at the Bedford College for Women on "Modern Methods of Fighting Disease." He first referred to the work of Pasteur on germs, and showed a series of slides exhibiting the forms of bacterial life associated with various diseases, and indicated the efforts that have been made to use anti-toxic sera, etc., in treatment. He gave an account of the steps by which the part played by insects as the intermediate host of the higher organisms of malaria, yellow fever, sleeping sickness, etc., have been ascertained, and of the methods which in consequence of that knowledge are being applied to stamp out the diseases which they transmit. He then told how Malta fever was traced to an organism inhabiting the goat and transmitted in its milk, and he showed how the number of cases of Malta fever in the garrison of that island have been reduced since goat's milk has ceased to be an article of diet among its members. He concluded with an account of how the connection was traced between the thyroid gland and myxœdema, and how that disease is successfully treated by thyroid extract.

---

At the request of her Majesty the Queen a Committee, of which Sir Frederick Treves is the head, has expended £1,000—being part of the sum realised by the sale of the Queen's Christmas Gift Book—in furniture, clothing, and appliances for presentation to the military hospitals of the United Kingdom. The articles include adjustable invalid couches and easy chairs, hand-wheel and rattan chairs, bed-tables, sick-room stoves, striped blankets (known as Guards' rugs), down pillows, dressing gowns, footstools, hot-water bottles, and reading lamps. Each bears on a small ivory tablet or linen badge the words : "The Gift of Queen Alexandra, 1909." They are on exhibition in the windows of Messrs. Shoolbred, Tottenham Court Road, who have supplied them.

Sir John Craggs having placed at the disposal of the London School of Tropical Medicine a fund for the encouragement of investigations into the various causes of tropical disease, grants from this fund have been awarded to Dr. R. Howard, of the Universities Mission to Central Africa, and Dr. B. M. Wilson, of Fiji.

---

The West African section of the London Chamber of Commerce gave a complimentary dinner at the Criterion Restaurant on March 18 to Professor W. J. H. Simpson, M.D., F.R.C.P., on his return from West Africa after his investigations, on behalf of the Colonial Office, into the existence of plague and the sanitary measures in force there. Sir Ralph Moor, chairman of the section, presided, and the company included Mr. Charles Charleton (chairman of the Council of the Chamber), Sir William Church, Sir Patrick Manson, F.R.S. (Medical Adviser to the Colonial Office), Sir Shirley Murphy and Sir Roper Parkington, and a number of ladies. Professor Simpson, in responding to the toast of his health, said that a nation which had abolished slavery, had extinguished tribal wars, and suppressed human sacrifice in West Africa, was quite capable of making that country healthy. His experience in West Africa had convinced him that malaria was not prevented because there was no organised service there to deal with the conditions favouring the disease. Any organisation must provide some security for continuity of policy and effort. For that purpose he proposed that there should be a sanitary commissioner, with a deputy commissioner, for each colony and dependency, in charge of the local medical officers, and responsible to the Government for the efficiency of the sanitary work. In the larger towns there should be medical officers of health entirely occupied with sanitary duties. Arrangements should be made for training West Africans in sanitary science. He had no doubt that a scheme of this sort would inaugurate a new era in West Africa, and malaria would become as rare there as it is in London.

---

### Women and the College of Surgeons.

The new by-law which is to come before the Council of the Royal College of Surgeons at their next meeting in April for final approval regarding the admission of women to examination for the diplomas of the College is in three sections, and is as follows : "(*a*) Pursuant to the powers conferred by the Medical Act of 1876, and subject to the provisions therein and hereinafter contained, women may be admitted as Members and Fellows of the College, and may obtain diplomas in dental surgery on the same terms and conditions as men, and so far as it is necessary to give effect to this by-law words in the by-laws and regulations of the College which import the masculine gender shall also import the feminine gender and all proper alterations shall be made in the form of the letters, testimonials, diplomas, certificates, and licenses granted by the College. (*b*) Women shall not be eligible as members of the council or vote at or take any part in any election of a member or members of the council, or attend any meeting of Fellows or of Fellows and Members (except meetings convened for the delivery of lectures or orations), or otherwise take any part in the government, management, or proceedings of the College. (*c*) Women shall not be eligible as members of the Court of Examiners or for any examinership to which the council appoint." We understand that next July is the earliest date at which women students, provided the above proposed by-law is approved, will be able to enter for the College examinations.

